# Supplementary material for: Repurposing Clemastine to Target Glioblastoma Cell Stemness
Source: Cancers (Basel). 2023 Sep 18;15(18):4619. doi: 10.3390/cancers15184619 (PMC10526458; doi:10.3390/cancers15184619)
Supplement: Supplementary file 1 [file cancers-15-04619-s001.zip › cancers-2582619-supplementary/File_S1.pdf]

File S1: Original Images for Blots and Gels

Figure 2B

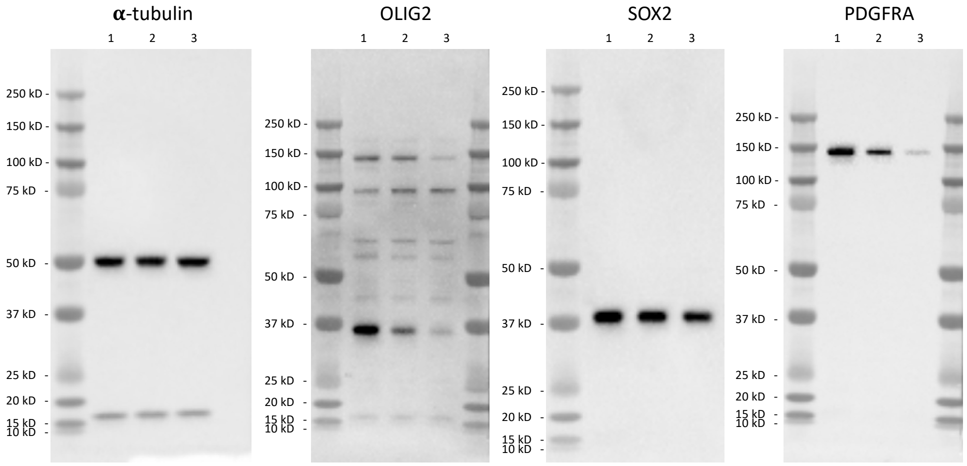

BTIC#102 13d treatment
Lane 1: Vehicle
Lane 2: Clemastine 2 µM
Lane 3: Clemastine 4 µM

Supplementary Fig. 3B

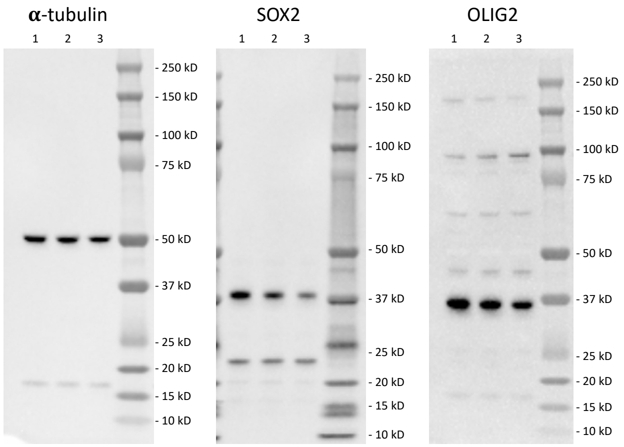

BTIC#148 10d treatment
Lane 1: Vehicle
Lane 2: Clemastine 2 µM
Lane 3: Clemastine 4 µM

Supplementary Fig. 6E

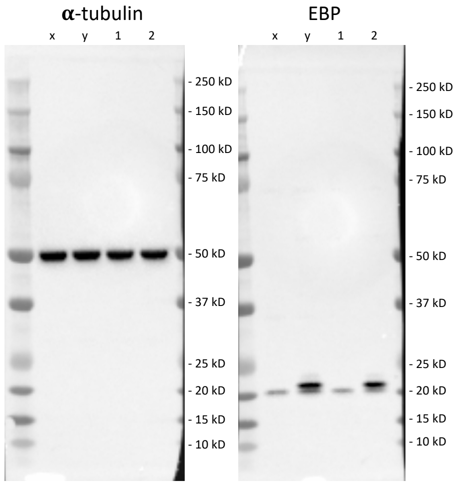

BTIC#102 *EBP* overexpression
Lane x: irrelevant
Lane y: irrelevant
Lane 1: pcDNA3.1-Ctl (vector control)
Lane 2: pcDNA3.1-*EBP* (*EBP*-overexpressed)

Supplementary Fig. 6G

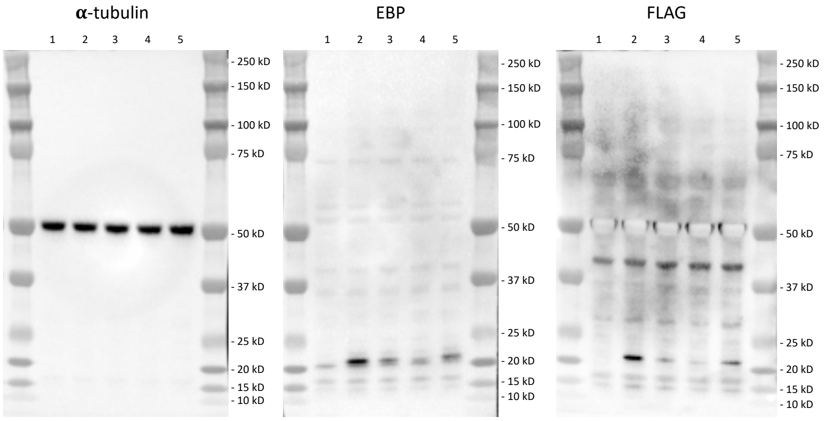

BTIC#102 WT/mutant EBP overexpression
Lane 1: pcDNA3.1-Ctl (vector control)
Lane 2: pcDNA3.1-*EBP*-WT (wild-type EBP)
Lane 3: pcDNA3.1-*EBP*-E80K (E80K EBP mutant)
Lane 4: pcDNA3.1-*EBP*-R147H (R147H EBP mutant)
Lane 5: pcDNA3.1-*EBP*-W196S (W196S EBP mutant)
