# Supplementary material for: Repurposing Clemastine to Target Glioblastoma Cell Stemness
Source: Cancers (Basel). 2023 Sep 18;15(18):4619. doi: 10.3390/cancers15184619 (PMC10526458; doi:10.3390/cancers15184619)
Supplement: Supplementary file 1 [file cancers-15-04619-s001.zip › cancers-2582619-supplementary/Supplementary Figures_S1-8.pdf]

Supplementary Figures

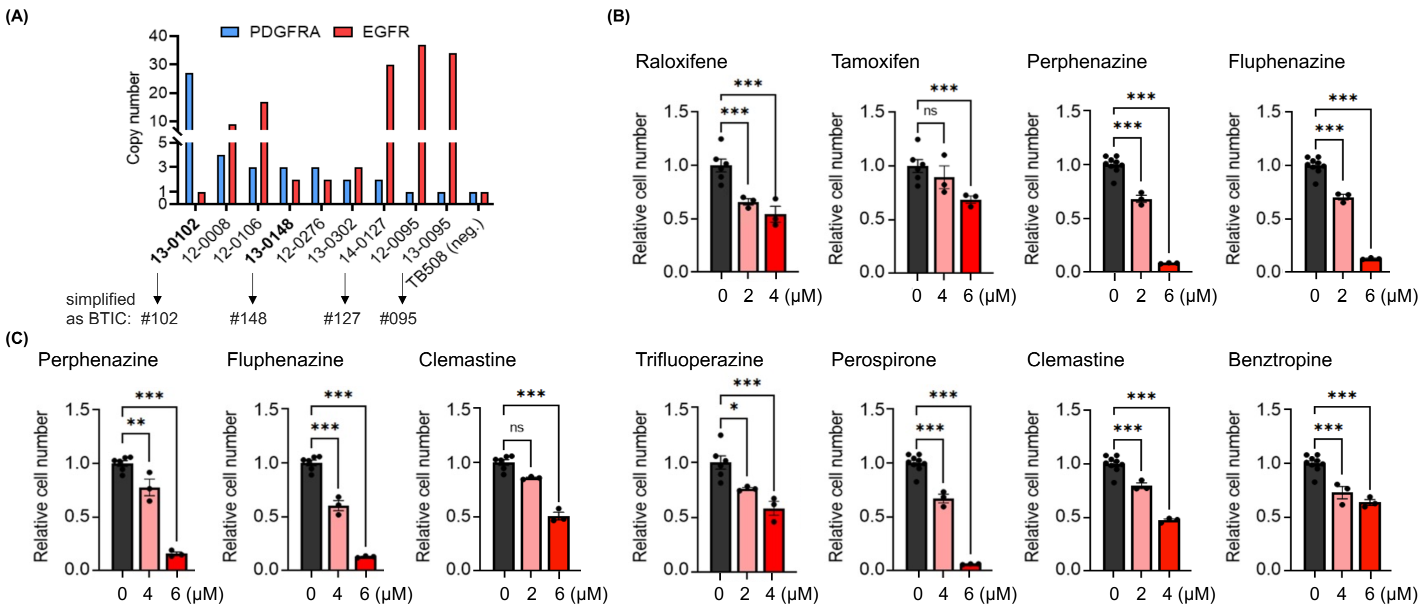

Figure S1

Figure S1. OPC-differentiating agents inhibit the proliferation of patient-derived BTIC cultures bearing *PDGFRA* amplification. (A) Normalized copy numbers of *PDGFRA* and *EGFR* genes in 9 patient-derived GBM cultures. Copy numbers of both genes are normalized to the copy numbers of *RNase P*. TB508 serves as a negative control cell line with a single copy of both *PDGFRA* and *EGFR* genes. Simplified IDs for GBM cultures used in this study were noted. **(B-C)** Quantification of relative cell proliferation of **(B)** BTIC#102 and **(C)** BTIC#148 cells treated with indicated OPC-differentiating agents and doses. The y-axis represents normalized phase area confluence at day 7-8 (normalized to day 0 and then normalized to respective vehicle controls). n = 6 for all vehicle groups except for the perospirone, perphenazine, fluphenazine, clemastine, and benztropine-treated BTIC#102 panels, n = 9. n = 3 for all remyelinating agents-treated groups. (B-C) Data are represented as mean ± S.E.M.. Significance was calculated using one-way ANOVA followed by Dunnett's multiple comparisons tests and represented as *p < 0.05, **p < 0.01, ***p < 0.001, n.s.: not significant.

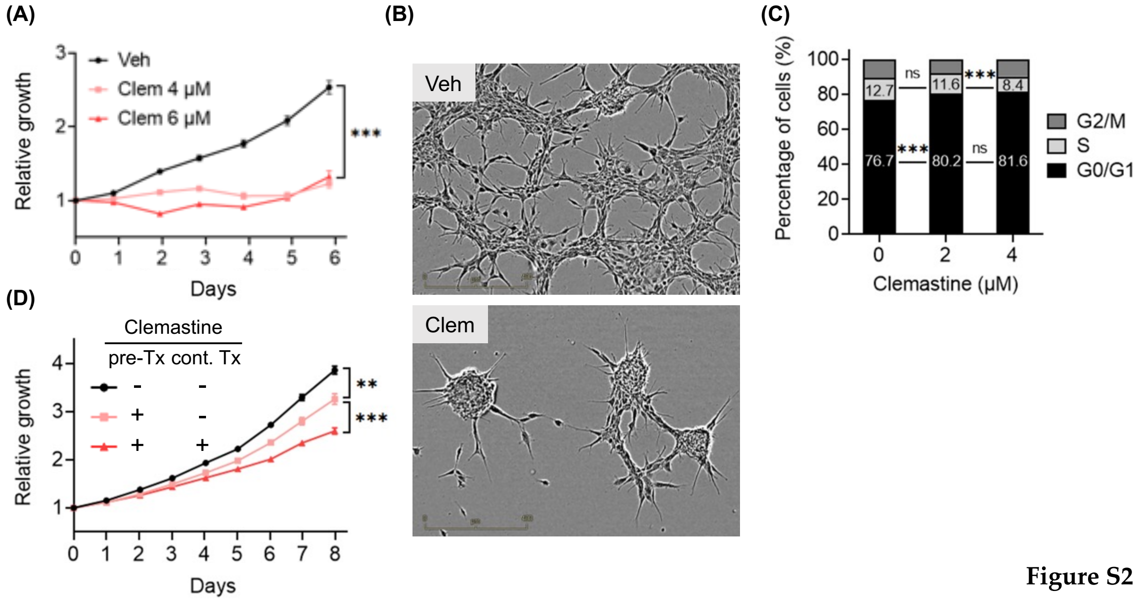

Figure S2

Figure S2. Clemastine suppresses the proliferation of patient-derived BTIC cultures bearing *PDGFRA* amplification. (A) Proliferation of BTIC#148 cells treated with clemastine (Clem) at indicated doses. n = 8 per condition except vehicle, n = 16. **(B)** Representative images (4x, scale bar: 400 µm) of BTIC#148 cells treated with vehicle (Veh) or clemastine (Clem; at 4 µM) for 9 days in laminin-coated plates. **(C)** Quantification of cell cycle analysis of BTIC#148 cells treated with clemastine at indicated doses for 16 days. n = 2 per condition. **(D)** Proliferation of BTIC#148 cells with or without 10-day clemastine (4 µM) pre-treatments (pre-Tx) and/or subsequent clemastine (4 µM) treatments (cont. Tx). Cell proliferation was monitored during subsequent clemastine treatments. n = 18 for pre-Tx^{-}cont.Tx^{-}, n = 9 for pre-Tx^{+}cont.Tx^{-}, and n = 6 for the pre-Tx^{+}cont.Tx^{+} group. “-“: no clemastine in the media; “+”: with clemastine in the media. (A, D) Data are represented as mean ± S.E.M.. Significance was calculated using two-way repeated measures ANOVA followed by (A) Dunnett’s or (D) Tukey’s multiple comparisons tests or (C) two-way ANOVA followed by Tukey’s multiple comparisons tests and represented as *$p < 0.05$, **$p < 0.01$, ***$p < 0.001$, n.s.: not significant.

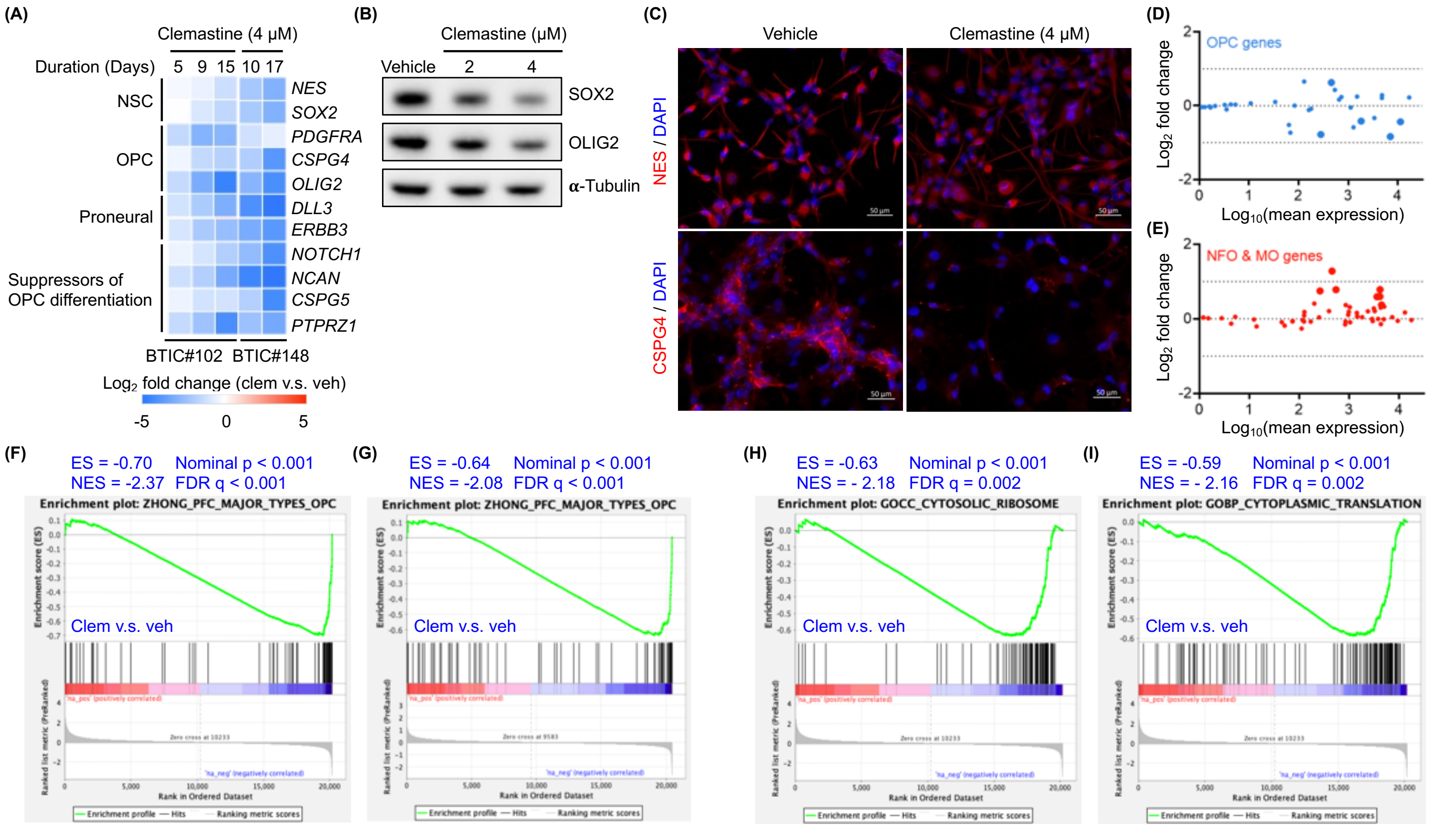

Figure S3

Figure S3. Clemastine attenuates the stemness and progenitor cell features of *PDGFRA*+ BTIC cells. (A) The heatmap of mRNA expression fold change (clemastine v.s. vehicle) of genes associated with NSCs, OPCs, proneural subtype GBM, and suppressors of OPC differentiation in BTIC#102 and BTIC#148 cells treated with clemastine (4 μM) for the indicated duration of time as assessed by quantitative RT-PCR. n = 3 replicates per condition. **(B-C)** Protein levels of NSC and OPC markers in BTIC#148 cells treated with clemastine at indicated doses for **(B)** 10 days or **(C)** 16 days as assessed by **(B)** immunoblot assays (representative results from two independent experiments are shown) or **(C)** IF staining (representative images from two independent experiments are shown; 20x, scale bar: 50 μm). **(D-E)** MA plots summarizing differential mRNA expression of top 40 **(D)** OPC-specific genes or **(E)** oligodendrocytes-specific genes between clemastine versus vehicle-treated (17-day treatment) BTIC#148 cells. Larger symbols indicate genes with adjusted p-values < 0.05. **(F-I)** GSEA plots comparing the transcriptomic profiles between clemastine (Clem) versus vehicle (Veh)-treated **(F, H-I)** BTIC#102 (15-day treatment) or **(G)** BTIC#148 (17-day treatment) cells with the following gene sets: **(F-G)** OPC, (**H**) cytosolic ribosome, and **(I)** cytoplasmic translation gene sets.

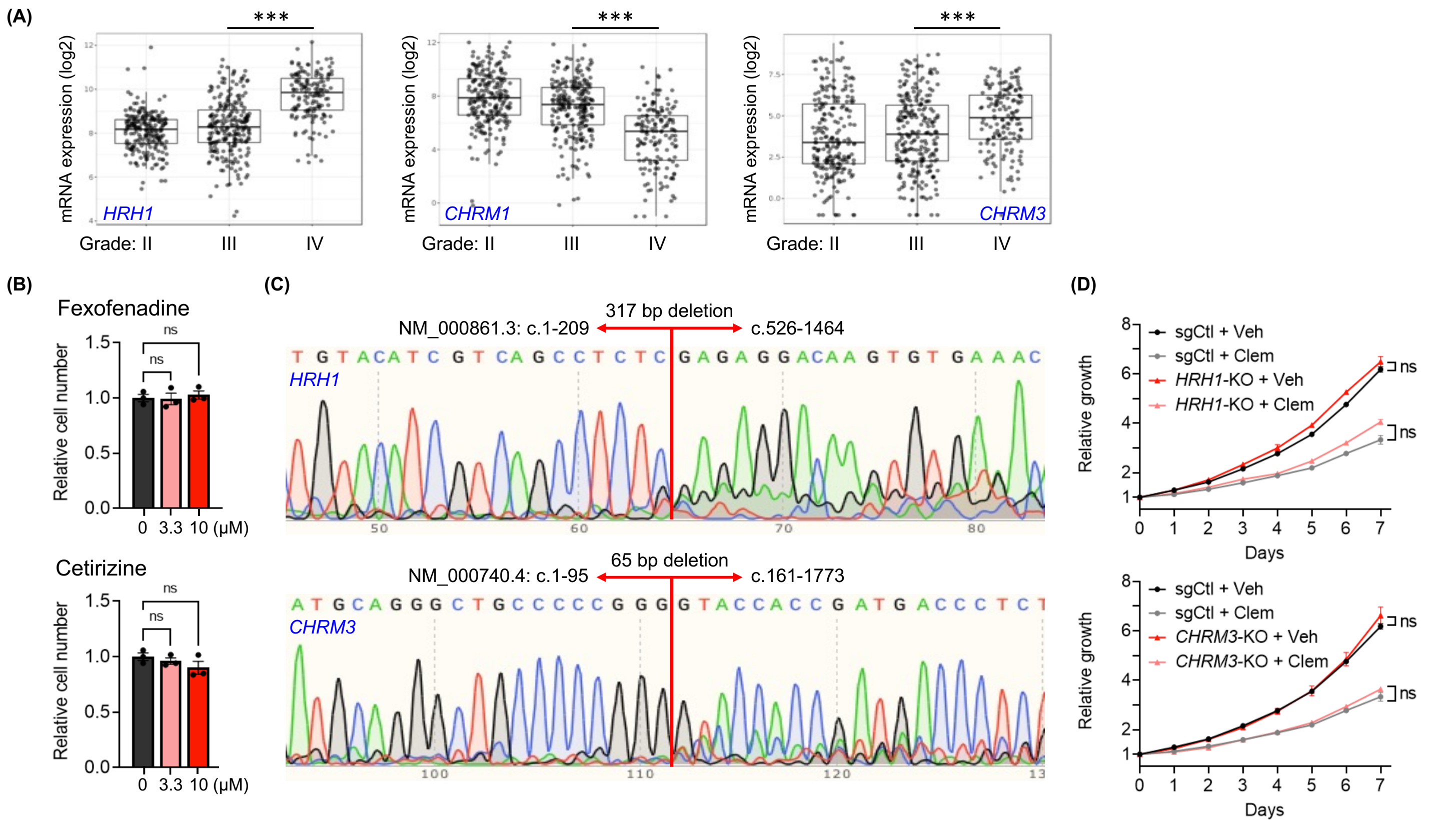

Figure S4

Figure S4. Assessing the roles of potential pharmacological targets of clemastine in BTICs. (A) Boxplots of mRNA expression levels of *HRH1*, *CHRM1*, and *CHRM3* in GBMs and low-grade gliomas (data retrieved from TCGA_GBMLGG dataset). Plots were generated using the GlioVis portal. **(B)** Quantification of relative cell proliferation of BTIC#102 cells treated with indicated H1R antagonists and doses. The y-axis represents normalized phase area confluence at day 7 (normalized to day 0 and then normalized to respective vehicle controls). n = 3 per condition. **(C)** Sanger sequencing results of the amplicon targeting the coding sequence of *HRH1* gene in *HRH1*-knockout BTIC#102 cells (upper panel) and *CHRM3* gene in *CHRM3*-knockout BTIC#102 cells (lower panel). The results indicate a 317 bp deletion in the coding region of the *HRH1* gene (c.210_525del) and a 65 bp deletion in the coding region of the *CHRM3* gene (c.96_160del). **(D)** Proliferation of non-targeting sgControl (sgCtl) and *HRH1*-knockout (*HRH1*-KO) BTIC#102 cells (upper panel) or *CHRM3*-knockout (*CHRM3*-KO) BTIC#102 cells (lower panel) treated with vehicle (Veh) or clemastine (Clem; at 4 μM). n = 3 per condition. (B, D) Data are represented as mean ± S.E.M.. Significance was calculated using (A) Tukey's HSD tests by the GlioVis portal, (B) one-way ANOVA followed by Dunnett's multiple comparisons tests, or (D) two-way repeated measures ANOVA followed by Tukey's multiple comparisons tests, and represented as *p < 0.05, **p < 0.01, ***p < 0.001, n.s.: not significant.

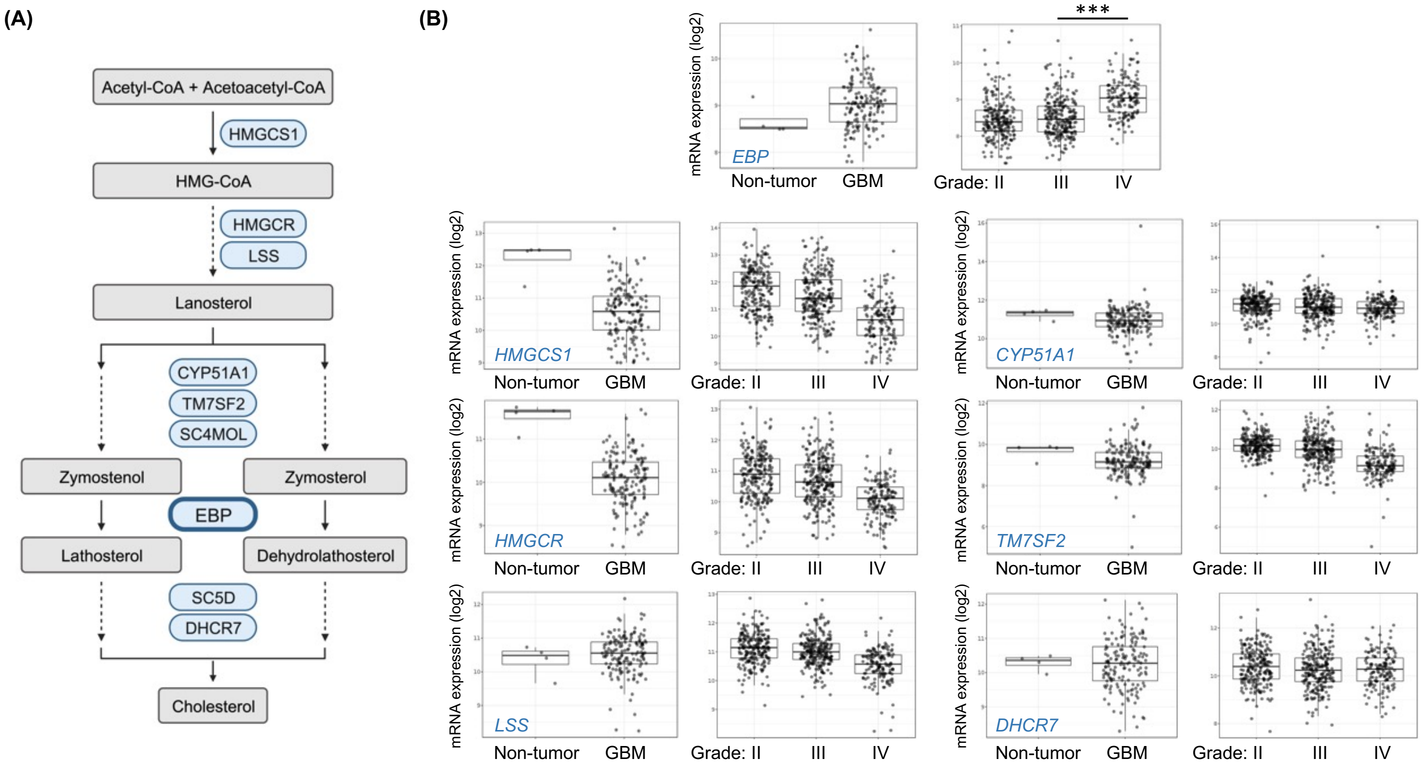

Figure S5

Figure S5. ***EBP*** **displays distinct expression patterns in gliomas in comparison to other genes in the cholesterol biosynthesis pathway. (A)** Schematic representation of sterol metabolites (in black) and enzymes (in blue) involved in the human cholesterol biosynthesis pathway. The figure was created with BioRender.com. **(B)** Boxplots of mRNA expression levels of genes in the cholesterol biosynthesis pathway (data for *SC4MOL* and *SC5D* were not available) in GBMs and non-tumors (left panel, data retrieved from TCGA_GBM dataset and RNA-seq platform) or low-grade gliomas (right panel, data retrieved from TCGA_GBMLGG dataset). Plots were generated using the GlioVis portal. Significance was calculated using Tukey's HSD tests by the GlioVis portal and represented as *p < 0.05, **p < 0.01, ***p < 0.001, n.s.: not significant.

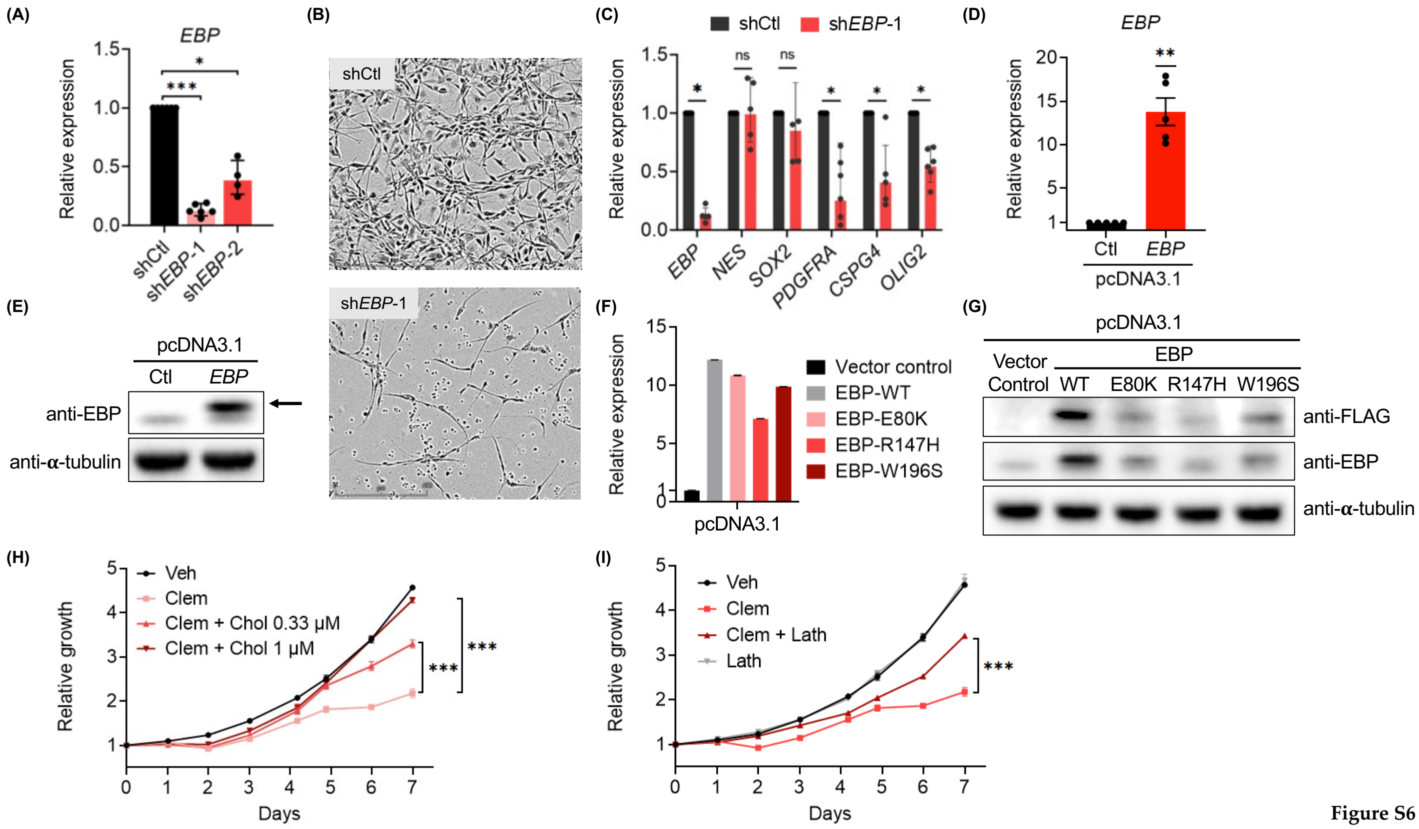

Figure S6. EBP is essential for BTIC propagation. (A) mRNA expression levels of *EBP* in shCtl, sh*EBP*-1, and sh*EBP*-2 BTIC#102 cells as assessed by quantitative RT-PCR. n = 6 per condition except sh*EBP*-2, n = 4. **(B)** Representative images (4x, scale bar: 400 µm) of shCtl and sh*EBP*-1 BTIC#148 cells 10 days after lentiviral transduction. **(C)** mRNA expression levels of genes associated with NSCs and OPCs in shCtl and sh*EBP*-1 BTIC#102 cells as assessed by quantitative RT-PCR. n = 5 per gene except *PDGFRA* and *OLIG2*, n = 6. **(D)** mRNA expression levels of *EBP* in vector control (pcDNA3.1-Ctl) and *EBP*-overexpressed (pcDNA3.1-*EBP*) BTIC#102 cells. n = 5 per condition. **(E)** Protein levels of EBP in vector control and *EBP*-overexpressed BTIC#102 cells as assessed by immunoblot assays (representative results from two independent experiments are shown). **(F)** mRNA expression levels of *EBP* (wild-type or mutant) in BTIC#102 cells with vector control, overexpression of wild-type EBP, or overexpression of each of the three mutants as assessed by quantitative PCR. n = 3 replicates per group. **(G)** Protein levels of FLAG-EBP (wild-type or mutant) in BTIC#102 cells with vector control, overexpression of wild-type EBP, or overexpression of each of the three mutants as assessed by immunoblot assays (representative results from two independent experiments are shown) using anti-FLAG and anti-EBP antibodies. **(H)** Proliferation of BTIC#148 cells treated with clemastine (Clem; at 6 µM) with or without water-soluble cholesterol (Chol) at indicated doses. n = 6 per condition. **(I)** Proliferation of BTIC#148 cells treated with clemastine (Clem; at 6 µM) and/or lathosterol (Lath; at 3.13 µM). n = 6 per condition. (A, C-D) Data are represented as geometric mean ± geometric S.D. of fold change relative to control groups, (F) geometric mean ± S.E.M., or (H-I) mean ± S.E.M.. Significance was calculated using (H-I) two-way repeated measures ANOVA followed by Tukey's multiple comparisons tests and represented as *p < 0.05, **p < 0.01, ***p < 0.001, n.s.: not significant.

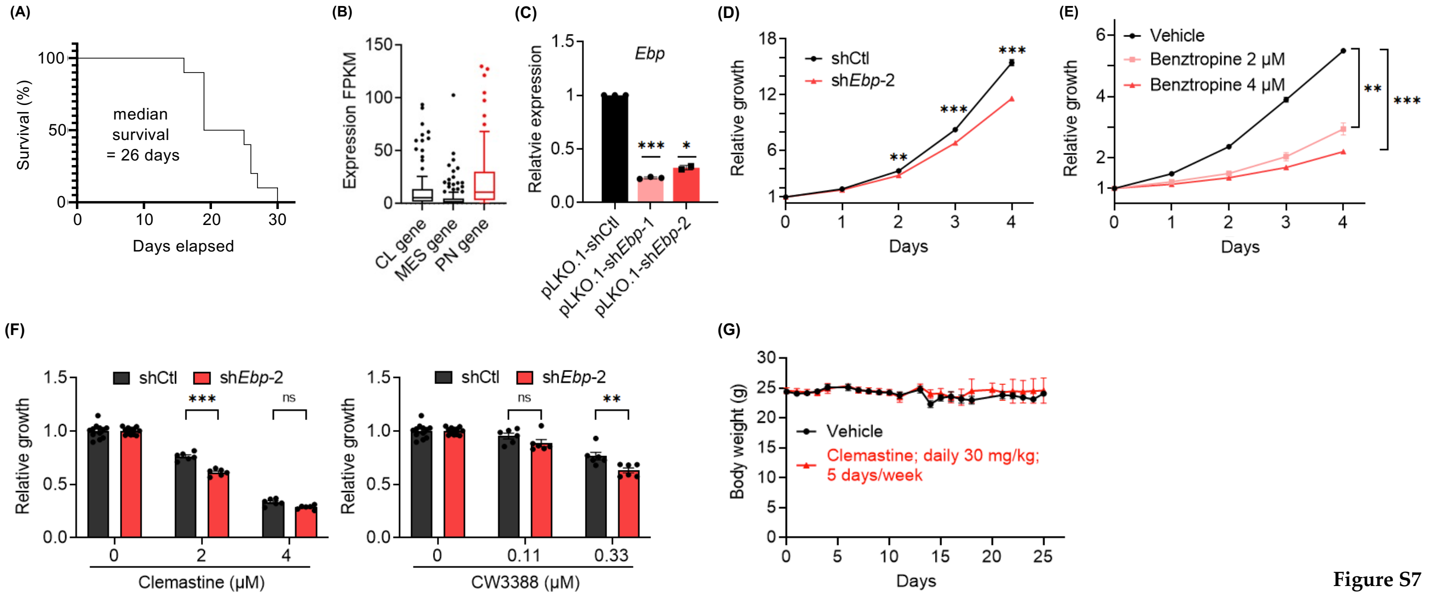

Figure S7

Figure S7. Loss of Ebp impairs the growth of C266 mouse glioma cells derived from p53-null mouse NSCs with PDGFB overexpression. (A) The Kaplan Meier analyses of mice orthotopically implanted with mouse p53-null NSCs transduced with PDGFB-overexpressing retrovirus. n = 10 mice. **(B)** Boxplots of mRNA expression levels (FPKM) of markers genes for classical (CL, n = 127), mesenchymal (MES, n = 174), or proneural (PN, n = 133) in mouse glioma tumors as assessed by mRNA-seq (only genes detected in the tumor samples and matched between mouse and human were used for the analysis. Note the intra-tumoral heterogeneity and the relative dominance of the PN subtype). An independent analysis using the human gene training set described in Ref. 84 yielded consistent subtyping results. **(C)** Quantitative RT-PCR analyses confirmed the reduced *Ebp* transcript levels induced by gene knockdown. n = 3 per condition except sh*Ebp*-2, n = 2. **(D)** Proliferation of shCtl and sh*Ebp*-2 C266 cells. P-values indicate significance between shCtl and sh*Ebp*-2 cells at indicated timepoints. n = 12 per condition. **(E)** Proliferation of C266 cells treated with benztropine at indicated doses. n = 6 per condition except vehicle, n = 12. **(F)** Quantification of relative cell proliferation of shCtl and sh*Ebp-2* C266 cells treated with clemastine (left panel) or CW3388 (right panel) at indicated doses. The y-axis represents normalized phase area confluence at day 4 (normalized to day 0 and then normalized to respective vehicle controls). n = 6 per condition except for the vehicle groups of the clemastine-treated panel, n = 9. **(G)** The average mouse body weight of *in vivo* orthotopic mouse models derived from C266 cells treated with vehicle (30% DMSO in PBS) or clemastine (30 mg/kg) five times per week. Day 0 on the x-axis indicates the treatment start date. n = 21 mice for the vehicle group and n = 20 mice for the clemastine-treated group. Data are represented as (C) geometric mean ± geometric S.D. of fold change relative to the shCtl group or (D-G) mean ± S.E.M.. Significance was calculated using two-way repeated measures ANOVA followed by (D) Sidak's or (E) Dunnett's multiple comparisons tests, or (F) two-way ANOVA followed by Sidak's multiple comparisons tests, and represented as *p < 0.05, **p < 0.01, ***p < 0.001, n.s.: not significant.

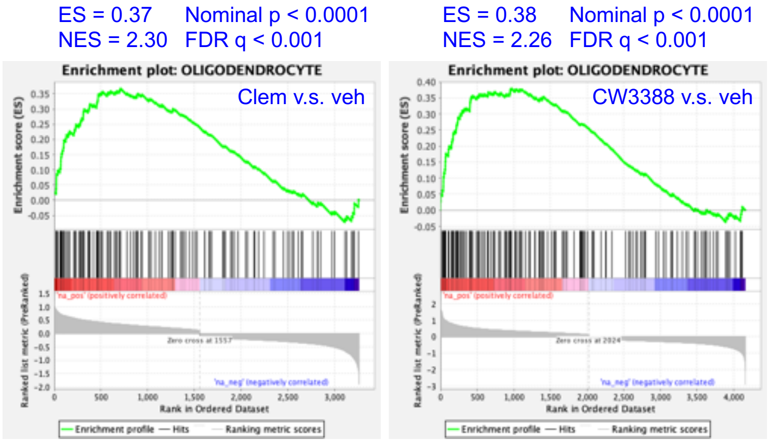

Figure S8

Figure S8. Both clemastine and CW3388 treatments upregulate the oligodendrocyte gene set in C266 mouse glioma cells. GSEA plots of genes differentially expressed between (left panel) clemastine (Clem) or (right panel) CW3388 versus vehicle (Veh)-treated C266 cells (12-day treatment) with the mouse oligodendrocyte gene set.
