# Supplementary material for: Repurposing Clemastine to Target Glioblastoma Cell Stemness
Source: Cancers (Basel). 2023 Sep 18;15(18):4619. doi: 10.3390/cancers15184619 (PMC10526458; doi:10.3390/cancers15184619)
Supplement: Supplementary file 1 [file cancers-15-04619-s001.zip › cancers-2582619-supplementary/Table_S1.pdf]

Table S1. List of primers, shRNA sequences, plasmids, chemicals, reagents, and antibodies.

Purposes	Gene name	Forward (F) or Reverse (R)	Sequence (5’ to 3’)
RT-qPCR	*ACTB*	F	GATTCCTATGTGGGCGACGA
		R	AGGTCTCAAACATGATCTGGGT
	B2M	F	TGCCGTGTGAACCATGTG
		R	ACCTCCATGATGCTGCTTACA
	NES	F	CTGGAGCAGGAGAAACAGGG
		R	GAGGGAAGTCTTGGAGCCAC
	SOX2	F	AACGGCAGCTACAGCATGAT
		R	GACTTGACCACCGAACCCAT
	PDGFRA	F	GACGGTCTTGGAAGTGAGCA
		R	TAAAGCCCTGTCTGCTGTCG
	CSPG4	F	GGGCCGTTCTCTATAGCCAC
		R	GACACCATCACCAGGTAGCC
	OLIG2	F	AGTGGCTTCAAGTCATCCTCG
		R	TGTTGATCTTGAGACGCAGCT
	DLL3	F	TAGCGCTCATTTTCCTCCCC
		R	TGGATCTGCAGCTCGAAGAC
	PTPRZ1	F	GCAGTTGGATGGAGAGGACC
		R	TCGACAATCAGTTGGTCGCT
	CSPG5	F	GAGCTTCTAGTGCCCACTGG
		R	CATCCCCTTGTGCCAGATGT
	NOTCH1	F	CAGATCCTGATCCGGAACCG
		R	CCAGAAACAGGGGTGTCTCC
	NCAN	F	GTGTCACTGCCTTCCTACCC
		R	TGCAATGATGGCTGAGCTGA
	MOG	F	AGGGAAAGGTGACTCTCAGGA
		R	GGAAGATGAGGCCAACAGTGA
	ERBB3	F	TGAATGGCCTGAGTGTGACC
		R	ATCGTAGACCTGGGTCCCTC
	EBP	F	TACTGGCTGGCCTCTTCTCT
		R	CCCAGGATGTATCGGCTGTC
	Actb	F	GGTGGGAATGGGTCAGAAGG
		R	GTACATGGCTGGGGTGTTGA
	B2m	F	TTTCAGTGGCTGCTACTCGG
		R	TGTTCGGCTTCCCATTCTCC
	Ebp	F	ATCGAGGGCTGGTTCTCTCT
		R	GCCCATAGACACCACAAGCT

Purposes	Name	Forward (F) or Reverse (R)	Sequence (5’ to 3’)
sgRNA	sg-*HRH1*-exon2-F1	F	CACCGTACATCGTCAGCCTCTCGG
	sg-*HRH1*-exon2-R1	R	AAACCCGAGAGGCTGACGATGTAC
	sg-*HRH1*-exon2-F2	F	CACCGCAGACCTCGGTGCGCCGAG
	sg-*HRH1*-exon2-R2	R	AAACCTCGGCGCACCGAGGTCTGC
	sg-*CHRM3*-exon5-F1	F	CACCGGGTCATCGGTGGTACCGTC
	sg-*CHRM3*-exon5-R1	R	AAACGACGGTACCACCGATGACCC
	sg-*CHRM3*-exon5-F2	F	CACCGAAATGAGTGACGGTTCCCG
	sg-*CHRM3*-exon5-R2	R	AAACCGGGAACCGTCACTCATTTC
	sgRNA-control-F	F	CACCGTAGGCGCGCCGCTCTCTAC
	sgRNA-control-R	R	AAACGTAGAGAGCGGCGCGCCTAC
PCR	PCR-*HRH1*-F1	F	GGTCACAGTAGGGCTCAACC
	PCR-*HRH1*-R1	R	CACCACCAGCATCTTTTGGC
	PCR-M13F-*CHRM3*-F1	F	TGTAAAACGACGGCCAGTCCCCCAGACTATGTCAGAGAG
	PCR-M13F-*CHRM3*-R1	R	CAGGAAACAGCTATGACCCTAAGGCCCATCGATTCATGA
	pLentiCRISPR-R1	N/A	CCCGTTGCGAAAAAGAACGT
EBP mutagenesis	*E80K*	F	CATTCACCTGGTGATCAAGGGCTGGTTCGTTCT
		R	AGAACGAACCAGCCCTTGATCACCAGGTGAATG
	R147H	F	GCCAGCATCCCCTCCACTTCATTCTACAGCT
		R	AGCTGTAGAATGAAGTGGAGGGGATGCTGGC
	W196S	F	GTCTTCATGAATGCCCTGTCGCTGGTGCTG
		R	CAGCACCAGCGACAGGGCATTCATGAAGAC
	QuikChange Primer Design Program: https://www.agilent.com/store/primerDesignProgram.jsp?_requestid=1072141		

Purposes	Gene name	Clone ID	Sequence (5’ to 3’)
shRNA	*EBP* #1 (human)	TRCN0000049393	CCGG-GCACCTAAGACTGGACAACTT-CTCGAG-AAGTTGTCCAGTCTTAGGTGC-TTTTTG
	EBP #2 (human)	TRCN0000049394	CCGG-CTCCGCTTCATTCTACAGCTT-CTCGAG-AAGCTGTAGAATGAAGCGGAG-TTTTTG
	Ebp #1 (mouse)	TRCN0000111870	CCGG-CCAGAAGACTCAAATCTTCTT-CTCGAG-AAGAAGATTTGAGTCTTCTGG-TTTTTG
	Ebp #2 (mouse)	TRCN0000111872	CCGG-CCAAGGGAGATAGCCGATATA-CTCGAG-TATATCGGCTATCTCCCTTGG-TTTTTG
	Non-targeting control	SHC002	CCGG-CAACAAGATGAAGAGCACCAA-CTCGAG-TTGGTGCTCTTCATCTTGTTG-TTTTTG
