# Supplementary material for: Repurposing Clemastine to Target Glioblastoma Cell Stemness
Source: Cancers (Basel). 2023 Sep 18;15(18):4619. doi: 10.3390/cancers15184619 (PMC10526458; doi:10.3390/cancers15184619)
Supplement: Supplementary file 1 [file cancers-15-04619-s001.zip › cancers-2582619-supplementary/Table_S2.pdf]

Table S2. List of plasmids, chemical compounds, reagents, kits, and equipment.

Category	Name	Vendor	Cat. Number
Plasmids	pcDNA3.1 V5-His A	GenScript	N/A
	pcDNA3.1$^+$ C-(K)-DYK-hEBP	GenScript	OHu18817
	pcDNA3.1$^+$ C-(K)-DYK-hEBP-E80K	This lab	N/A
	pcDNA3.1$^+$ C-(K)-DYK-hEBP-R147H	This lab	N/A
	pcDNA3.1$^+$ C-(K)-DYK-hEBP-W196S	This lab	N/A
	EGFP-hGal3	Addgene	73080
	PM-GFP	Addgene	21213
	LentiCRISPRv2E	Addgene	78852
Chemical compounds	Clemastine (fumarate)	Cayman	14637
	Lathosterol	Cayman	9003102
	Cholesterol-water soluble	Sigma-Aldrich	C4951
	Tamoxifen	Sigma-Aldrich	T5648
	Puromycin dihydrochloride	Sigma-Aldrich	P8833
	Dimethyl sulfoxide	Sigma-Aldrich	D2650-100ML
	Ascorbic acid	Lonza	CC-4398
	Geneticin (G418 Sulfate)	ThermoFisher	10131035
	CW3388	Sigma-Aldrich	SML2602
	Polybrene	Sigma-Aldrich	TR-1003
	TASIN-1	Cayman	21155
	Cetirizine	Cayman	19686
	Fexofenadine	Cayman	18191
	D-luciferin, sodium salt	Goldbio	LUCNA-1
	2-Mercaptoethanol	Sigma-Aldrich	M7522-100ML
	Tween 20	BIO-RAD	1706531
	Methanol	VWR	BDH1135-4LP
	Ethanol absolute	VWR	89125-172
	Isoflurane	Patterson	07-893-8441
	DAPI	MilliporeSigma	D9542-10MG
	Fluphenazine (hydrochloride)	Cayman	23555
	Perphenazine	Cayman	20735
	Trifluoperazine (hydrochloride)	Cayman	15068
	Raloxifene (hydrochloride)	Cayman	10011620
	Benztropine (mesylate)	Cayman	16214
	Perospirone (hydrochloride)	Selleck	S4889
	Saponin	MilliporeSigma	84510-100G
Reagents and kits	Human PDGF-AA	Shenandoah	100-16
	Human PDGF-BB	Shenandoah	100-18
	Laminin	MilliporeSigma	L2020
	Triton X-100 solution	Fluka	93443
	Formalin 10%, neutral buffered	VWR	89370-094
	Pierce™ 16% Formaldehyde (w/v), Methanol-free	ThermoFisher	28908
	Ponceau S solution	Sigma-Aldrich	P7170
	Nitrocellulose Membrane, 0.45um; Roll 30 cm x 3.5 m	Bio-Rad	1620115
	Trans-Blot Turbo RTA Mini 0.2 µm Nitrocellulose Transfer Kit	Bio-Rad	1704270
	NuPAGE 4-12% Bis-Tris Protein Gels, 1.0mm, 12 well	ThermoFisher	NP0322BOX

	NuPAGE 4-12% Bis-Tris Protein Gels, 1.0mm, 10 well	ThermoFisher	NP0321BOX
	NuPAGE 4-12% Bis-Tris Protein Gels, 1.5mm, 15 well	ThermoFisher	NP0336BOX
	NuPAGE 4-12% Bis-Tris Protein Gels, 1.5mm, 10 well	ThermoFisher	NP0335BOX
	Mini Gel Tank	ThermoFisher	A25977
	RNA to cDNA EcoDry™ Premix (Double Primed)	Takara (Clontech)	639548
	KAPA SYBR FAST qPCR Master Mix (2X)	Roche	KK4602
	SuperSignal™ West Pico PLUS Chemiluminescent Substrate	ThermoFisher	34580
	Pierce™ BCA Protein Assay Kit	ThermoFisher	23225
	Accutase solution	MilliporeSigma	A6964-500ML
	Corning 96-well Clear Flat Bottom Polystyrene TC-treated Microplate	Corning	3595
	Quick-RNA miniprep kit	Genesee	11-328 (Zymo R1055)
	UltraPure DNase/RNase-Free Distilled Water	ThermoFisher	10977015
	Dulbecco's Phosphate Buffered Saline	MilliporeSigma	D8537-500ML
	PBS, pH 7.4	ThermoFisher	10010023
	DPBS, no calcium, no magnesium	ThermoFisher	14190144
	4X Laemmli Sample Buffer	BIO-RAD	1610747
	Trypan Blue Solution (0.4%)	Sigma-Aldrich	T8154
	Opti-MEM™ I Reduced Serum Medium	ThermoFisher	31985070
Reagents and kits	McCoy's 5A (Modified) Medium	ThermoFisher	16600082
	Trypsin (2.5%), no phenol red	ThermoFisher	15090046
	Lipofectamine 3000 Transfection Reagent	ThermoFisher	L3000008
	Bovine Serum Albumin	Sigma-Aldrich	A9647-100G
	Restore Western Blot Stripping Buffer	ThermoFisher	21059
	NuPAGE MOPS SDS Running Buffer (20X)	ThermoFisher	NP0001
	NuPAGE Antioxidant	ThermoFisher	NP0005
	Corning 1L 10X Tris Buffered Saline	Corning	46-012-CM
	10x Tris Buffered Saline (TBS)	BIO-RAD	1706435
	ProLong Gold Antifade Mountant	ThermoFisher	P36934
	SlowFade Diamond Antifade Mountant	ThermoFisher	S36972
	FxCycle™ PI/RNase Staining Solution	ThermoFisher	F10797
	S.O.C. Medium	ThermoFisher	15544034
	TempPlate full-skirted 96-well PCR plate	USA Scientific	1402-9800
	Precision Plus Protein Dual Color Standards	BIO-RAD	1610374
	50x TAE (Tris/Acetic Acid/EDTA) Buffer, 1 L	BIO-RAD	1610743
	Non-fat dry milk	Genesee	20-241
	Goat serum	MilliporeSigma	G9023-10ML
	QuikChange Lightning Multi Site-Directed Mutagenesis Kit	Agilent	210519
	ZymoPURE Plasmid Miniprep Kit	Genesee	11-553
	Neon Transfection System 10 µL Kit	ThermoFisher	MPK1025
	Bioruptor Standard	Diagenode	UCD-200
	CFX96 Real-Time PCR Detection System	BIO-RAD	N/A
	Nunc Lab-Tek II Chamber Slide System - 2-well Chamber Slide w/ removable wells	ThermoFisher	154461
Equipment	3-well removeable chamber slides	Ibidi	80381
	PowerPac Basic Power Supply	BIO-RAD	1645050
	Trans-Blot Turbo Transfer System	BIO-RAD	1704150
	ChemiDoc MP System	BIO-RAD	1708280
