# Supplementary material for: Repurposing Clemastine to Target Glioblastoma Cell Stemness
Source: Cancers (Basel). 2023 Sep 18;15(18):4619. doi: 10.3390/cancers15184619 (PMC10526458; doi:10.3390/cancers15184619)
Supplement: Supplementary file 1 [file cancers-15-04619-s001.zip › cancers-2582619-supplementary/Table_S3.pdf]

Table S3. List of antibodies used for immunoblotting and immunofluorescence staining.

Name	Vendor	Catalog Number	RRID	Dilution for immunoblotting	Dilution for IF
Nestin	MilliporeSigma	ABD69	AB_2744681		1:500
SOX2	Cell Signaling	3579S	AB_2195767	1:1000	1:400
PDGF receptor **α**	Cell Signaling	3174S	AB_2162345	1:1000	
PDGF receptor **α**	Cell Signaling	5241S	AB_10692773		1:400
CSPG4	Cell Signaling	43916S	N/A		1:200
OLIG2	Novus	NBP1-28667	AB_1914109	1:3000	1:1000
Galactocerebroside	MilliporeSigma	MAB342	AB_94857		1:200
EBP	Santa Cruz	sc-374267	AB_11012162	1:500	
**α**-tubulin	Cell Signaling	3873S	AB_1904178	1:1000	
Anti-rabbit IgG, HRP-linked Antibody	Cell Signaling	7074S	AB_2099233	1:1000	
Anti-mouse IgG, HRP-linked Antibody	Cell Signaling	7076S	AB_330924	1:1000	
Goat anti-Rabbit IgG (H+L) - Alexa Fluor 594	ThermoFisher	A-11037	AB_2534095		1:500
Goat anti-Rabbit IgG (H+L) - Alexa Fluor 647	ThermoFisher	A-21245	AB_2535813		1:500
Goat anti-Rabbit IgG (H+L) - Alexa Fluor Plus 488	ThermoFisher	A32731	AB_2633280		1:500
Goat anti-Mouse IgG (H+L) - Alexa Fluor 488	ThermoFisher	A-11029	AB_2534088		1:500
Goat anti-Mouse IgG (H+L) - Alexa Fluor Plus 488	ThermoFisher	A32723	AB_2633275		1:800-1:1000
Goat anti-Mouse IgG (H+L) - Alexa Fluor Plus 647	ThermoFisher	A32728	AB_2633277		1:200-1:1000
Normal Rabbit IgG	MilliporeSigma	12-370	AB_145841		1:1200-1:20000
Normal Mouse IgG	MilliporeSigma	12-371	AB_145840		1:2500
Rabbit IgG Isotype Control	ThermoFisher	31235	AB_243593		1:1200-1:20000
Mouse IgG Isotype Control	ThermoFisher	31903	AB_10959891		1:2500
